# Deviation of the Fecal Stream in Colonic Bowel Segments Results in Increased Numbers of Isolated Lymphoid Follicles in the Submucosal Compartment in a Novel Murine Model of Diversion Colitis

**DOI:** 10.1155/2017/5265969

**Published:** 2017-08-13

**Authors:** Annabel Kleinwort, Paula Döring, Christine Hackbarth, Claus-Dieter Heidecke, Tobias Schulze

**Affiliations:** ^1^Department of General Surgery, Visceral, Thoracic and Vascular Surgery, Universitätsmedizin Greifswald, Greifswald, Germany; ^2^Institute of Pathology, Universitätsmedizin Greifswald, Greifswald, Germany

## Abstract

**Introduction:**

Diversion colitis is a significant health problem due to its high incidence in patients with diverting enterostomy. This mucosal inflammation presents characteristic histopathological features allowing for the differentiation of this entity from other inflammatory bowel diseases. The pathophysiology of this disease remains ill-defined, in part due to the lack of appropriate animal models. The present study was performed in order to develop and characterize a murine model of diversion colitis.

**Methods:**

A diverting loop colostomy was performed in C57BL/6 mice either in the ascending colon or in the transverse colon. Animals were assessed for clinical and histopathological parameters during short-term and long-term survival.

**Results:**

Animals with a colostomy in the transverse colon showed a good long-term survival and developed a mild colitis in the bypassed bowel closely resembling the human pathology on a histopathological level.

**Conclusion:**

This model is a promising tool to further elucidate the pathomechanism leading to impaired mucosal homeostasis in bypassed colonic segments. Moreover, the establishment of the model in the C57BL/6 background allows the combination of this colitis model with various transgenic mouse strains to investigate the effect of locally deregulated mucosal immunity on systemic immune homeostasis and to develop specific therapeutic strategies.

## 1. Introduction

Diversion colitis is an inflammation of colon segments excluded from the fecal stream. The term was coined by Glotzer et al. in 1981 in a clinical report on patients without prior inflammatory bowel disease who developed inflammation of the excluded colon segments after ileostomy or colostomy [1]. Morson and Dawson have described a similar disease as early as in the 1970s [2]. Diversion colitis develops in 50% to 91% of patients with diverting enterostomy [3–5]. Disease severity is mild in about half of the affected patients; 44% suffer from moderate and 4% from severe inflammatory activity [3]. In the face of estimated 0.7 million colostomy patients and an annual incidence of 120,000 patients in the United States of America, diversion colitis represents a frequent clinical problem significantly deteriorating the quality of life of affected patients [5].

Human deviation colitis shows a broad spectrum of histopathological changes. These are more pronounced in the distal than in the proximal sections of the diverted bowel. The most common histologic findings comprise mild chronic inflammation, architectonical changes of the crypts (distortion, dilatation, and atrophy), and lymphoid follicular hyperplasia. The latter are considered as hallmark lesions of deviation colitis by the majority but not by all authors [3, 6–8]. In some cases, aphthoid ulcerations can be associated with lymphoid nodules. Surface epithelial degeneration with reduced cell height, cytoplasmic eosinophilia, and nuclear pyknosis have been described [6]. Crypt abscesses may occur but are uncommon. Intramucosal loose granulomas are sometimes formed in close relation with ruptured cysts [7]. Ulcerations, inflammatory pseudomembranes, and hemorrhagic necrosis are uncommon [1]. The inflammatory infiltrate is mostly constituted of plasma cells and lymphocytes and to a lesser extent of macrophages and eosinophils [9, 10]. The inflammatory changes are predominantly localized in the mucosa.

Although a considerable amount of clinical research was performed in the 1990s and early 2000s to clarify the cellular and molecular mechanisms triggering and maintaining the inflammation in the excluded bowel segments, no satisfying pathophysiological concept covering all aspects of the disease has been established yet. Multiple mechanisms have been proposed, including the overgrowth with a single pathogenic agent, the local production of toxic metabolites, and a modification of the local microenvironment with deleterious consequences for colonocyte metabolism and mucosal perfusion [7, 10]. Although it was demonstrated that the composition of the bacterial flora is altered to the disadvantage of obligate anaerobes in bypassed human colonic segments, neither a single pathogenic organism nor a toxic metabolite has been isolated so far [7, 11]. The changes in the intestinal flora might lead to a local decrease of fermentation products, for example, short fatty acids, leading to trophic impairment of colonocytes and local ischemia [12]. However, small clinical trials with the local substitution of short fatty acids or their precursors have shown inconsistent results [13, 14]. Moreover, immune dysregulation has been proposed to be implicated in the pathogenesis of diversion colitis, as anti-inflammatory treatment regimens have been found to ameliorate diversion colitis in experimental models [15, 16]. However, the exact mechanisms remain undefined.

The investigation of the immunopathological mechanisms implicated in the pathogenesis of diversion colitis as well as the consequences of the smoldering mucosal inflammation on the systemic immune homeostasis is severely hampered by the lack of an appropriate murine model. Diversion colitis has been successfully induced in rats by creating Hartmann's colostomy or an end colostomy with mucus fistula [15, 17, 18]. Although these models reliably reproduce many histological characteristics of human diversion colitis, a murine disease model offers several advantages. Most importantly, numerous genetically modified mouse models deficient for typical mediators implicated in T_H_1 and T_H_17 dominated immune pathologies are available to determine the role of these molecules in the pathogenesis of diversion colitis. Furthermore, there are numerous well-characterized murine models of typical T_H_1, T_H_2, and T_H_17 dominated immune responses that can be combined with a murine model of diversion colitis to assess the influence of chronic colonic mucosal inflammation on the systemic immune homeostasis. In this paper, we describe for the first time a murine model of diversion colitis with special emphasis on the kinetics of disease onset and development as well as on its histopathological characterization.

## 2. Methods and Material

### 2.1. Animals

Fourteen-week-old male C57BL/6 mice purchased from Charles River, Germany, were held under SPF-conditions, adapting for at least two weeks before experimentation. Since gender impacts on the severity of colitis in various colitis models as well as human disease, only animals of one sex were used in this study. As intestinal inflammation is more pronounced in male animals in chemically induced colitis models, male animals were chosen to establish the model of diversion colitis [19]. Starting from one week preoperatively, mice were fed with Sniff M-Z Ereich (V1184-3) to obtain high body weights (at least 25 g). Mice had free access to water and food. The animal experiments were approved by the veterinary government authority (Landesamt für Landwirtschaft, Lebensmittelsicherheit und Fischerei Mecklenburg-Vorpommern, LALLF M-V). All efforts were made to minimize the number of animals used and their suffering. Animals were randomly assigned to 3 experimental groups: proximal colostomy (39 animals), distal colostomy (42 animals), and sham group (29 animals).

### 2.2. Surgical Procedures

**Figure 1:**
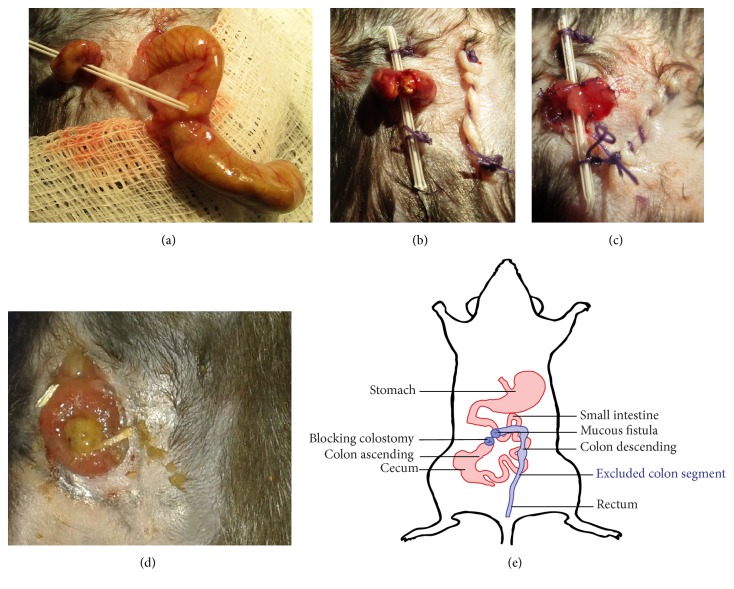
*Surgical procedure.* The photographic images show a distal colostomy operation. (a) The cecum is exteriorized after median laparotomy to identify the transverse colon. The transverse colon is pulled through a little incision in the right upper quadrant of the abdominal wall and stabilized by a small flexible tube passed through the mesocolon. (b) The cecum is transferred back into the peritoneal cavity and the laparotomy is closed in two layers. The flexible tube is sutured to the skin to avoid slipping of the exteriorized colon. The colon is opened by subtotal transection. (c) The afferent and efferent part of the colostomy are sutured to the skin, creating a blocking loop colostomy. (d) The blocking loop colostomy shows no signs of irritation after two months. (e) Graphical representation of the postoperative anatomy.

Surgery was performed under general anesthesia with ketamine (Selectavet Dr. Otto Fischer GmbH, Germany; 87 mg/kg i.p.) and xylazine hydrochloride (Xylazin, Bayer, Germany; 13 mg/kg i.p.). Mice received perioperative antibiotic prophylaxis with 25 mg/kg body weight ceftriaxone (Cephasaar, Germany) and 12.5 mg/kg body weight metronidazole (Braun Melsungen AG, Germany). All mice were operated by the same surgical team. Mice were placed in supine position and fixed with tape to ensure a stable position of the animal during the operation. After superficial shaving, all mice underwent a 15 mm median laparotomy. After identification of the cecal pole, the mobile colon ascending and transversum were mobilized out of the peritoneal cavity with the help of a DeBakey atraumatic forceps in order to allow for the unequivocal identification of the future colostomy position in the ascending colon (5 mm distal from Bauhin's valve, proximal colostomy (PC)) and the transverse colon (20 mm distal from the Bauhin's valve, distal colostomy (DC)). A small incision (3 mm) was made in the upper right quadrant. The previously identified colon segments were then exteriorized through the incision in the upper right quadrant. The position of the exteriorized loop was secured with a flexible tube (22 G, Braun, Germany; Figure 1(a)) passed through the mesocolon perpendicularly. Care has to be taken not to damage mesenteric vascular structures. The laparotomy was closed in two layers (aponeurosis and skin) of continuous suture (Vicryl Plus, polyfil 4-0 1/2c, Ethicon, Germany). The exteriorized colonic loop was opened by subtotal transection using fine scissors (Figure 1(b)). The colostomy openings were fixed to the skin with full-thickness sutures of PDS II (monofil 6-0 3/8c, Ethicon, Germany) in a way that a blocking loop colostomy was created (Figure 1(c)). In the sham group, the ascending colon was opened by subtotal transection 1 cm distal to the ileocecal valve and immediately closed with PDS II (monofil 6-0 3/8c, Ethicon, Germany). The laparotomy was closed as described above. Postoperatively, all groups received 20 ml/kg body weight 0.9% sodium chloride solution i.p. (NaCl, Braun). Postoperative analgesia was started by injecting 0.1 mg/kg body weight buprenorphine s.c. (Buprenovet® 0.3 mg/ml, Bayer, Germany).

### 2.3. Postoperative Treatment

In the first postoperative week, mice received drinking water supplemented with 1 mg/ml tramadol (Tramal® 100 mg/ml, Grünenthal, Germany). A Solid Drink Pad (SDSHPF-75 Dehyprev Vit BIO TripleATrading, Tiel, Netherlands) was supplied in the cage to compensate for decreased fluid intake due to reduced mobility. The mice were weighed and scored every day during the first week, every second day during the first month, and every third day during the second month.

### 2.4. Assessment of Postoperative Complications

Deceased animals were immediately subjected to necropsy. “Ileus” and “anastomotic leakage” were diagnosed on necropsy. “Stoma complications” were defined as peristomal abscess and mucocutaneous separation. “Wasting syndrome” was defined as incessant weight loss greater than 33% of the initial body weight in the absence of stoma complications and without evidence of anastomotic leakage and ileus at necropsy.

### 2.5. Disease Severity Score

In order to assess the influence of fecal diversion on the general health status, the clinical status of the experimental animals was assessed daily using a disease severity score as described elsewhere [20, 21]. Briefly, the disease score includes the following items: physical appearance (normal fur = 0; slightly ruffled fur = 1; moist, ruffled fur = 2; moist, ruffled fur with mucous eye discharge = 3); respiration (normal = 0; tachypnea = 1; dyspnea = 2; shallow breathing = 3); weight loss (<10% = 0; <20% = 1; <33% = 2; >33% = 3); spontaneous behaviour (normal = 0; reduced, slowed locomotion = 1; unsteady gait, kyphotic posture = 2; lateral position = 3); elicited behaviour (normal = 0; escape behaviour triggered by physical approach = 1; escape behaviour triggered by physical contact = 2; no escape behaviour = 3) and abdominal palpation (normal = 0; abdomen soft with slight pain reaction = 1; distended abdomen = 2; distended abdomen with severe tenderness = 3). The total clinical score was obtained by addition of the item scores for each time point. Mice were sacrificed by cervical dislocation two weeks, one month, or two months postoperatively.

### 2.6. Tissue Preparation for Histological Analysis

Colon and rectum were removed after sacrifice. The colon was flushed with ice-cold PBS to remove any fecal content. The length and weight of the colon distal from the colostomy/colotomy were determined in the colostomy group and the sham group, respectively. The colon weight/length ratio (CW/*L* ratio) was determined. The explanted colon was divided into three parts: colon segment bearing the colostomy/incision, distal colon, and rectum. The parts were cut into 5 mm long pieces. Tissue samples were formalin fixed, dehydrated, paraffin embedded, and sectioned horizontal (2 *μ*m) with a microtome. The sections were stained with hematoxylin and eosin, periodic acid Schiff (PAS) reaction for a demonstration of goblet cells, and chloroacetate esterase reaction for a demonstration of neutrophil granulocytes.

### 2.7. Histological Analysis

Histology was evaluated by an experienced GI pathologist (P.D.). Histology of the colon sections was analyzed by light microscopy (Nikon Eclipse Ci-L, Nikon Instruments Europe BV, Düsseldorf, Germany). Three histological parameters were quantified: (1) the number of lymphoid follicles per colon cross section (HE staining), (2) absolute crypt length (PAS reaction), and (3) the proportion of goblet cell-bearing crypt length as well as the absolute goblet cell number per crypt (PAS reaction). At least five mucosal crypts per mouse for the crypt lengths, at least nine mucosal crypts per mouse for absolute goblet cell numbers, and at least two cross sections of the diverted colon per mouse for the number of lymphoid follicles were evaluated and the arithmetic mean was calculated. Measurements of crypt length were performed with the NIS-Element BR4 software (Nikon Instruments Europe BV, Düsseldorf, Germany).

### 2.8. Statistical Analysis

Statistical analysis and graphs were performed with the GraphPad Prism software (GraphPad Software, Inc., La Jolla, USA). The groups were tested for Gaussian distribution with the Shapiro-Wilk-test. The colostomy groups and their corresponding sham groups were compared with Student's *t*-test when they were normally distributed; otherwise the Mann–Whitney *U* test was used. For multiple comparisons of the different time points of colostomy duration (14, 30, and 60 days), a one-way ANOVA or Kruskal-Wallis test was performed. For comparison of categorical variables, Fisher's exact test for two-sided analysis of up to 6 × 6 contingency tables was applied. *p* values < 0.05 were considered significant. In the figures, *∗* confirms *p* < 0.05 and ^*∗∗*^*p* < 0.01. The graphs show the mean and standard error when being normally distributed and the median and interquartile range otherwise.

## 3. Results

### 3.1. Surgery and Peri- and Postoperative Mortality

**Figure 2:**
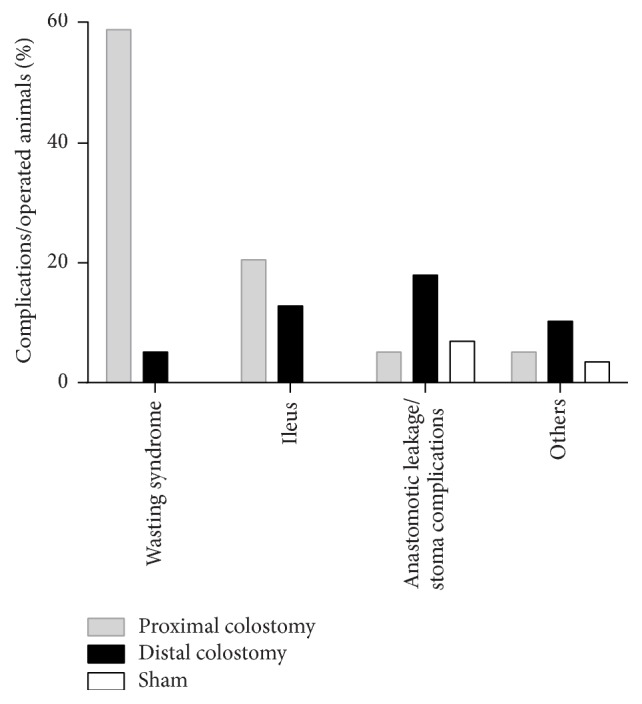
*Causes of death.* Postoperative complications were determined by necropsy.* Wasting syndrome* was defined as continuous weight loss greater than 33% of the initial body weight and no other findings on necropsy.* Ileus* and* anastomotic leakage* were diagnosed on necropsy.* Stoma complications* were defined as peristomal abscess and mucocutaneous separation. There were significant differences in the distribution of postoperative complications in between all groups (comparison of all groups: *p* < 0.001; comparison of only PC and DC: *p* < 0.001; comparison of only PC and sham: *p* < 0.001; comparison of only DC and sham: *p* = 0.021), analyzed by Fisher's exact test for two-sided analysis of up to 6 × 6 contingency tables. Animal numbers were 39 in the PC and DC group, respectively, and 29 in the sham group.

Surgery was well tolerated in all experimental groups. Intraoperative mortality was 0%, 0%, and 7.1% in the sham, proximal colostomy (PC), and distal colostomy (DC) group, respectively. Perioperative deaths were due to anesthesia side effects. In the PC group, irreversible weight loss (wasting syndrome) occurred in the early postoperative period until 100% of the animals had to be euthanized because they met the exclusion criteria of 33% weight loss. Mortality in the DC and sham group was 46.1% and 10.3%, respectively. Causes of death were wasting syndrome, ileus, or anastomotic leakage/stoma complications (Figure 2). Distribution of postoperative complications was significantly different between the three groups (*p* < 0.001).

### 3.2. Clinical Findings

**Figure 3:**
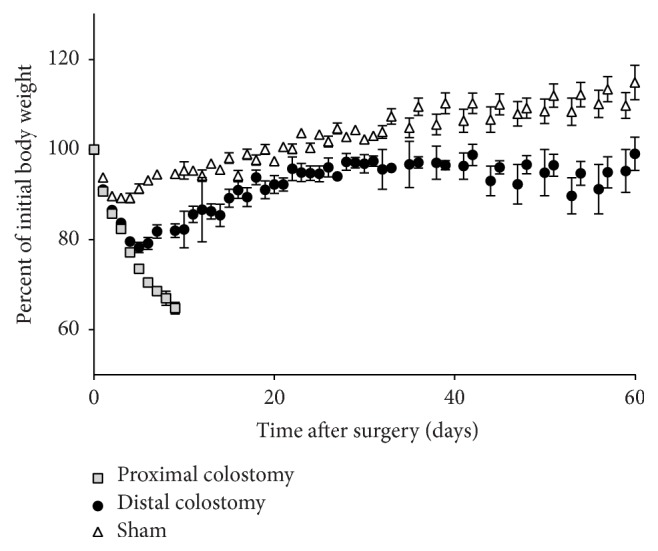
*Body weight development.* Body weight was measured daily. The body weight is shown as percentage of the preoperative body weight. Values are mean ± standard error of the mean of 21–35 animals per group (PC *n* = 35; DC *n* = 21; sham *n* = 26).

**Figure 4:**
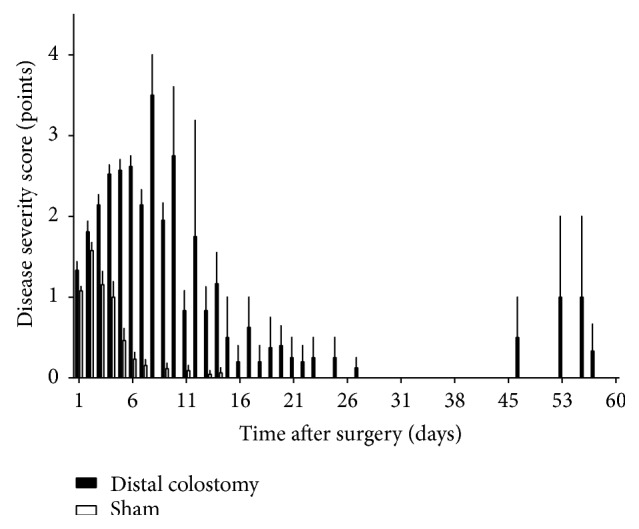
*Disease severity score.* A disease severity score including physical appearance, respiration, weight loss, spontaneous behaviour, elicited behaviour, and abdominal palpation was assessed to survey the influence of fecal diversion on the general health status. The graphic shows the scores of surviving animals in the DC and sham group. Values are mean and standard error of the mean of 21–26 animals per group (DC *n* = 21; sham *n* = 26).

During the early postoperative period, animals in all three experimental groups suffered a high weight loss that was significantly more pronounced in the PC and DC group than in the sham group (*p* = 0.0001) (Figure 3). While mice in the sham group attained the weight nadir on postoperative day 4 with a mean weight of 89.2% of the initial body weight, DC animals reached their minimal weight on postoperative day 5. Their mean weight loss was 21.7% of the initial body weight and thus significantly more pronounced than in the sham group (*p* < 0.0001). After the weight nadir in the first postoperative week, the weight of surviving animals rose constantly. Weight gain was more pronounced in the sham group than in the DC group (*p* = 0.0001) (Figure 3). Interestingly, although significant differences in the disease severity score between sham and DC animals occurred during the first month postoperatively, the scores were similar during the second postoperative month (Figure 4). Clinical signs of severe intestinal inflammation, that is, bloody discharge or liquid bowel movements, occurred neither in the sham nor in the DC group. In the DC group, the colostomy did not show overt signs of inflammation (Figure 1(d)). However, in some animals, swelling of the ipsilateral inguinal lymph node occurred.

### 3.3. Colon Weight/Length Ratio

**Figure 5:**
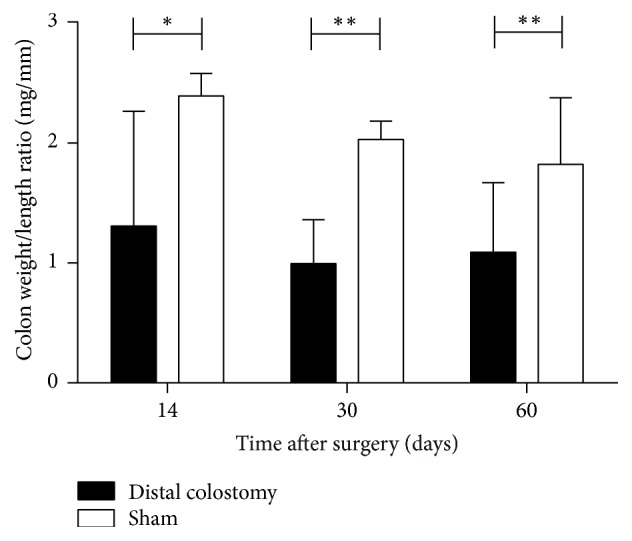
*Colon weight/length ratio.* In the DC group, there was a significant decrease of the CW/*L* ratio compared to sham at all investigated time points (^*∗*^*p* < 0.05; ^*∗∗*^*p* < 0.01). Values are median with interquartile range of 5–9 animals per group (DC 14 and 30 days, sham 14 days: *n* = 8; DC 60 days: *n* = 5; sham 30 and 60 days: *n* = 9).

After euthanasia at the chosen time points CW/*L* ratio was determined as described above. There was a significant decrease of the CW/*L* ratio in the DC group compared to the sham group at all investigated time points (*p* = 0.038 (14 days); *p* = 0.008 (30 days); *p* = 0.007 (60 days)) (Figure 5).

### 3.4. Histological Analysis

#### 3.4.1. Crypt Length

**Figure 6:**
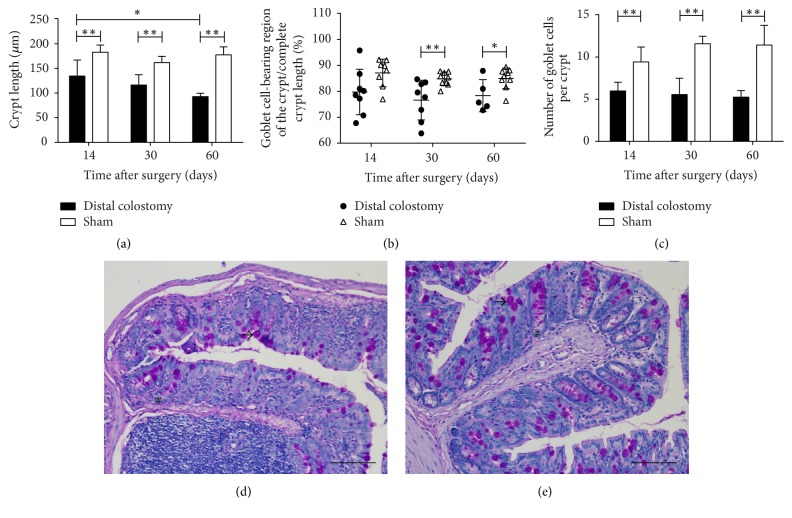
*Crypt length and goblet cells.* Crypt length and goblet cell numbers were determined on paraffin sections of the rectum after periodic acid-Schiff staining (PAS). (a) Crypt length was significantly decreased in the rectum of the DC group animals. (b) The length of the goblet cell-bearing region of the crypt was measured and set in ratio to the full crypt length. Thirty and sixty days postoperatively, the percentage of crypt length bearing goblet cells was decreased in the DC group. (c) Goblet cell numbers were significantly reduced in DC animals compared to the sham group. Graphs in (a)–(c) show the mean and standard error of 5 to 9 animals per group (DC 14 and 30 days, sham 14 days: *n* = 8; DC 60 days: *n* = 5; sham 30 and 60 days: *n* = 9); ^*∗*^*p* < 0.05; ^*∗∗*^*p* < 0.01. (d) and (e) Representative examples of sections of the rectum of DC (d) and sham (e) animals 30 days postoperatively stained with periodic acid Schiff. Crypt length (#) and goblet cells (→) can easily be identified.* Scale bars *represent 100 *μ*m.

Crypt length was significantly decreased in the colon of the DC group animals. Already after 14 days, crypt length in DC animals was 134.5 ± 32.6 *μ*m compared to 182.8 ± 14.7 *μ*m in the sham group (*p* = 0.002) (Figure 6(a)). After 30 days, the crypt length in the DC group was 116.4 ± 20.7 *μ*m compared to 162 ± 12.4 *μ*m in the sham group (*p* = 0.0001). After 60 days, crypt length in the DC group measured 92.9 ± 6.7 *μ*m compared to 177.6 ± 16.3 *μ*m in the sham group (*p* = 0.0001). The difference between the 14-day value and the 60-day value in the DC group was significant (*p* = 0.008), while crypt length in the sham group remained constant. Besides crypt shortening, no architectural changes like crypt branching were observed.

#### 3.4.2. Goblet Cells

Reduced goblet cell numbers are a histological feature of colonic segments devoid of fecal transit [22]. In order to characterize goblet cell quantity and distribution, the length of the goblet cell-bearing region of the crypt was measured and set in ratio to the complete crypt length. The percentage of crypt length bearing goblet cells was slightly decreased in the colostomy group. In the sham group, goblet cells were present in 84.8 ± 2.6% of the crypt length after 30 days and in 85.0 ± 4.0% of the crypt length after 60 days with higher density in the upper part of the crypt and absence in the crypt basis. In the colostomy group, the crypt length proportion containing goblet cells was slightly reduced to 76.6 ± 7.7% of the crypt length in the 30 days group (*p* = 0.009) and 78.3 ± 6.2% in the 60 days group (*p* = 0.03) (Figure 6(b)). The enumeration of absolute goblet cell number per crypt showed an absolute reduction of goblet cells in all DC group animals. After 14 days, there were 5.9 ± 1.1 goblet cells per crypt in the DC group compared to 9.4 ± 1.8 in the sham group (*p* = 0.0003). After 30 days, there were 5.6 ± 2 goblet cells per crypt in the DC group compared to 11.6 ± 0.9 in the sham group (*p* < 0.0001). After 60 days, the number of goblet cells was reduced to 5.3 ± 0.8 cells per crypt in the DC group compared to 11.4 ± 2.4 in the sham group (*p* = 0.001) (Figure 6(c)).

#### 3.4.3. Lymphoid Follicles

**Figure 7:**
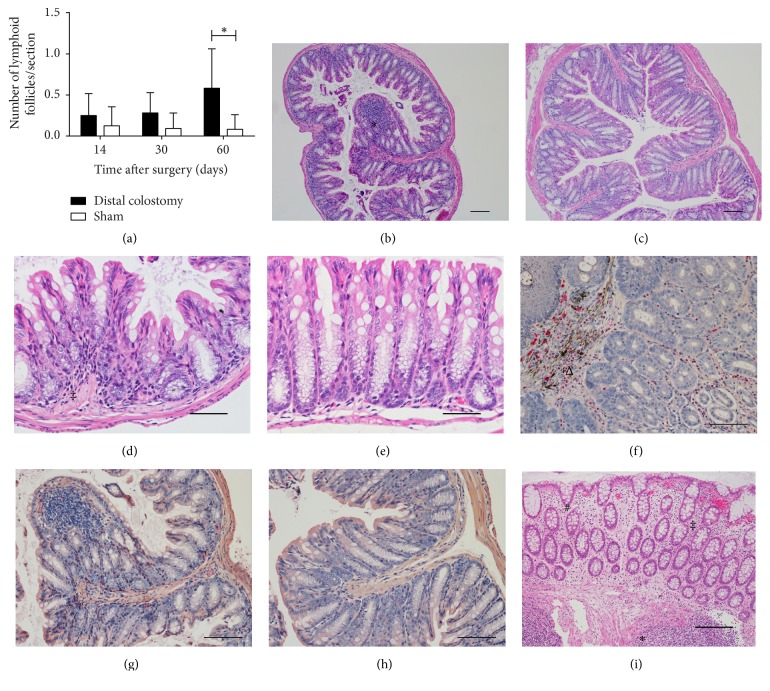
*Lymphoid follicles and inflammatory infiltrate.* (a) Lymphoid follicles of the diverted colon and sham animals were counted on paraffin sections stained with hematoxylin and eosin. The graph shows the mean and standard error of 5 to 9 animals per group (DC 14 and 30 days, sham 14 days: *n* = 8; DC 60 days: *n* = 5; sham 30 and 60 days: *n* = 9). (b) and (c) Representative examples of sections made of the rectum of DC (b) and sham (c) animals 60 days postoperatively stained with hematoxylin and eosin. A prominent lymphoid follicle (*∗*) is present in the mucosa of the DC mouse.* Scale bars *represent 100 *μ*m. (d) and (e) HE-stained sections of the rectum showed a slight hypercellularity (‡) due to inflammatory mononuclear cells in the lamina propria of DC animals (d) compared to the sham group (e).* Scale bars *represent 50 *μ*m. (f), (g), and (h) Staining with chloroacetate esterase reaction was performed to detect neutrophil granulocytes. A representative section of the vicinity of the colostomy opening illustrates an influx of neutrophils (Δ) 60 days postoperatively (f). On cross sections of the diverted rectum, an acute inflammatory neutrophilic infiltrate was observed neither in the DC group (g) nor in sham animals (h) up to 60 days postoperatively.* Scale bars *represent 100 *μ*m. (i) Human diversion colitis is characterized by a mild chronic inflammatory hypercellularity (‡) and edema of the lamina propria, slightly irregular crypts (#), and an increased number of enlarged lymphoid follicles (*∗*). No epithelial erosions were observed.* Scale bars *represent 100 *μ*m.

In the lamina propria of DC animals, a trend to the development of increased numbers of lymphoid follicles in the diverted rectum was observed 14 and 30 days postoperatively (*p* = 0.33 and *p* = 0.095, resp.). Sixty days postoperatively, this difference reached statistical significance (*p* = 0.014). In the DC group, there were 0.58 ± 0.48 lymphoid follicles per rectal cross section while in the sham group 0.08 ± 0.18 follicles were found (Figures 7(a), 7(b), and 7(c)).

#### 3.4.4. Surface Epithelium

Surface epithelium was evaluated after HE staining and classified as normal or eroded/ulcerated by an experienced GI pathologist. There was no evidence of surface erosions or ulcerations of the epithelium in the colostomy groups up to 60 days postoperatively, but luminal debris of apoptotic epithelial cells was slightly increased in DC animals.

#### 3.4.5. Inflammatory and Epithelial Reaction

In DC animals, a discrete hypercellularity by inflammatory mononuclear cells (lymphocytes) in the lamina propria was observed (Figures 7(d) and 7(e)). The influx of neutrophil granulocytes was only observed in the vicinity to the colostomy opening in individual animals (Figures 7(f), 7(g), and 7(h)). Epithelial regeneratory activity was slightly increased with elevated nuclear/cytoplasmic ratio, coarse nuclear chromatin, and increased basophilic staining of cytoplasma compared to sham animals.

## 4. Discussion

Diversion colitis is frequent in patients with dysfunctioned bowel segments. The incidence has been described to vary between 50% and 91% [3–5]. Although the majority of patients do not suffer from clinical symptoms, up to 40% of affected patients report clinical symptoms including mucous discharge, abdominal pain, bleeding, and tenesmus [1, 5, 23]. Severe clinical courses have been described sporadically [3, 24, 25]. However, symptoms can persist even after reversal of fecal diversion and negatively affect the quality of life [5, 23, 26]. In patients where reestablishment of intestinal continuity is not feasible, surgical resection of the dysfunctioned bowel segment may be necessary when medical anti-inflammatory treatment fails. Given the high prevalence of ostomy patients, diversion colitis remains a significant health issue.

This manuscript describes for the first time a murine model of diversion colitis. Ideally, an animal model for the human disease should be endowed with a close resemblance to the human pathology concerning histopathological changes, triggering factors, signs and symptoms, and clinical course. In order to facilitate further studies, it should have a well-defined genetic background and, if inflammation is an essential part of the pathology, a well-characterized immune system. The availability of antibodies for flow cytometry, ELISA, and Western blot substantially facilitates further analysis of the involvement of deregulated immune mechanisms in the pathogenesis. Murine disease models for intestinal inflammation offer these advantages.

In the murine model of diversion colitis presented here, we used a surgical procedure similar to that used in human disease to create colon segments excluded from fecal transit. The histopathological changes observed in the excluded colon segments strongly resembled those observed both in human diversion colitis and in rat models of diversion colitis. The hallmark lesion of human diversion colitis, pronounced lymphoid follicular hyperplasia, was present in virtually all animals after 60 days of stool diversion [6, 27]. Interestingly, Pacheco et al. also described the occurrence of lymphoid follicular hyperplasia as a histological characteristic in their rat model of diversion colitis [15]. Shortening of crypt length has been reported in both human and rat diversion colitis [9, 15, 17]. Keli et al. reported a reduction of crypt length of approximately 24% after six weeks and 42% after 17 weeks of fecal diversion. In our model, we observed a reduction of crypt length of 26% after two weeks, 28% after four weeks, and 48% after eight weeks. Our findings show that shortening of colonic crypts in bowel segments excluded from the fecal stream is an early event in the development of diversion colitis in our murine model. In our murine model, both the absolute number of goblet cells and the length of goblet cell-bearing crypt sections were reduced after fecal diversion. This is in accordance with observations made in rat models of diversion colitis [15, 17]. Goblet cell loss seems to be an early event during the development of diversion colitis since a significant reduction of goblet cell numbers could be observed as early as 14 days postoperatively. Since the goblet cell-free proportion of the crypt basis contains regenerative cells, the observed relative extension of this zone may indicate an increased epithelial regenerative activity after colostomy. Interestingly, some investigators proposed lymphoid follicles to promote mucosal repair via the regulation of bone-marrow derived stem cell trafficking [28, 29]. The observation of the correlation between increased lymphoid follicular hyperplasia and increased regenerative activity in colonic crypts deserves further exploration.

The lymphomononuclear infiltrate in diverted colon was moderate. This corresponds to observations made in rat models of diversion colitis [17]. However, in contrast to human diversion colitis, other rat models presented with a pronounced neutrophil infiltration indicating acute inflammation [18]. In human diversion colitis, lymphocytes are the main constituents of the mucosal infiltrate which can be moderate in colon segments without chronic inflammatory diseases prior to exclusion [10].

In our model, no evidence for surface erosions and ulcerations of the epithelium was observed. However, at least in a rat model, structural changes of the surface epithelium seem to be a late event [17]. In human diversion colitis, surface ulcerations are an inconstant finding [9, 30]. In conclusion, structural lesions of the surface epithelium seem to be of limited value in the assessment of murine diversion colitis, at least at early postoperative time points.

The colon weight/length ratio was significantly reduced in the excluded bowel segments. Increased colon weight/length ratios are considered a reliable marker of intestinal inflammation and damage in animal colitis models with severe inflammatory damage and gross inflammatory infiltrates [31]. They are mainly reported in inducible colitis models and adoptive transfer models showing high severity of inflammatory destruction. In the model described here, histopathological changes were characterized mainly by the reduction of crypt length and development of a lymphoid follicular hyperplasia. This is in accordance with observations made in a rat model of diversion colitis, showing a significant weight loss concerning the mucosa and muscularis propria in colon segments excluded from the fecal stream. Four weeks after colostomy, weight loss was as high as 35% in the lamina propria mucosae and 31% in muscularis propria [32]. These observations and our results suggest that the weight gain due to the inflammatory infiltrate and vascular congestion is compensated by the weight loss secondary to mucosal and muscular atrophy. Therefore, colon weight/length ratio seems not to be an appropriate tool to assess colonic inflammation in our disease model.

Although the inflammatory changes seen in the excluded bowel segments were moderate, we observed increased neutrophil and thrombocyte numbers in the blood (data not shown) indicating systemic effects of the smoldering lymphocytic colonic inflammation. Granulocytosis and thrombocytosis are paraclinical findings almost systematically observed in rodent colitis models not related to fecal diversion and in human inflammatory bowel disease [33]. These systemic paraclinical changes indicate that local mucosal inflammation in the excluded colon segments impacts on the immune homeostasis of the entire organism. Our model presents a well-characterized immune system and thus offers the possibility of further examining the impact of localized colonic inflammation on mucosal immunity in nondiverted segments of the intestinal tract and on the innate and adaptive immune response in various disease models.

Although clinical signs and symptoms of colitis were mild in our disease model and clinical disease scores were similar in sham and DC animals after the early postoperative period, the postoperative increase of body weight was significantly slower in DC animals than in the sham group. Though, feeding behavior was similar in the sham and DC group after the immediate postoperative period. Several mechanisms may account for this observation. Sustained inflammatory activation of the immune system may result in an increased catabolism. Furthermore, we cannot exclude that the presence of a colostomy interferes with the physiological coprophagy normally seen in small rodents. Up to 10% of their own fecal matter is consumed by rats [34]. However, while prevention of coprophagy results in vitamin deficiencies under conditions of limited access to nutrients, it loses its nutritional significance under experimental conditions with free access to water and nutritionally balanced food [35]. Finally, fecal diversion has been shown to result in modifications of the microbiome in the excluded bowel segments [17]. Changes in the composition of the intestinal microbiome have been reported to provoke alterations of the metabolism of nutrients affecting the energy balance by multiple mechanisms. First, they affect the host's capacity to absorb energy from otherwise indigestible food components. Second, modifications of the intestinal microbiome have an impact on the production of signaling molecules affecting the host energy metabolism [36, 37]. Whether limited local changes of the microbiome, for example, in bowel segments excluded from the fecal stream, are sufficient to induce the described systemic metabolic effects has not yet been investigated.

A major drawback of our model is the high mortality during the initial postoperative period. In the PC group, mortality attained 100%. Mortality occurred early in the postoperative course. The main cause of mortality in the PC group was weight loss exceeding the limit defined as an exclusion criterion. Given the early postoperative occurrence of this phenomenon, mortality most probably reflects high fluid and nutrient losses due to the localization of the colostomy adjacent to the ileocecal valve. Thus, long-term survival after proximal colostomy adjacent to the ileocecal valve seems not possible in mice. The incidence of ileus and local colostomy complications which were responsible for 27.7% and 38.9% of early postoperative deaths in the DC group, respectively, may be reduced by improvement of surgical techniques. However, the cause of the wasting syndrome accounting for 11.1% of early mortality in DC animals remained undetermined. Although feeding behavior of these animals appeared normal and, on autopsy, no signs of ileus or other surgical complications were detectable, animals continuously lost weight until exclusion criteria were met. Further metabolic and histopathological analyses are required at earlier time points in order to determine the pathomechanisms leading to this clinical picture.

In conclusion, the murine model of diversion colitis reproduces a significant number of histopathological and clinical features of the human disease. This model is thus a promising tool to determine the immunopathological mechanism triggering diversion colitis, to assess the interaction between the mucosal immune system and the microenvironment in excluded bowel segments and to test potential local and systemic therapeutic strategies. The combination of our model with other murine models for T_H_1-, T_H_2-, or T_H_17-polarized immune responses may provide a promising tool to assess the impact of diversion colitis on systemic immune homeostasis.

## References

[1] Glotzer D. J., Glick M. E., Goldman H. (1981). Proctitis and colitis following diversion of the fecal stream. *Gastroenterology*.

[2] Morson B., Dawson I. (1972). *Gastrointestinal Pathology*.

[3] Whelan R. L., Abramson D., Kim D. S., Hashmi H. F. (1994). Diversion colitis - A prospective study. *Surgical Endoscopy*.

[4] Haas P. A., Fox T. A., Szilagy E. J. (1990). Endoscopic Examination of the Colon and Rectum Distal to a Colostomy. *The American Journal of Gastroenterology*.

[5] Son D. N., Choi D. J., Woo S. U. (2013). Relationship between diversion colitis and quality of life in rectal cancer. *World Journal of Gastroenterology*.

[6] YEONG M. L., BETHWAITE P. B., PRASAD J., ISBISTER W. H. (1991). Lymphoid follicular hyperplasia–a distinctive feature of diversion colitis. *Histopathology*.

[7] Edwards C. M., George B., Warren B. (1999). Diversion colitis - New light through old windows. *Histopathology*.

[8] Roe A. M., Warren B. F., Brodribb A. J. M., Brown C. (1993). Diversion colitis and involution of the defunctioned anorectum. *Gut*.

[9] Geraghty J. M., Talbot I. C. (1991). Diversion colitis: Histological features in the colon and rectum after defunctioning colostomy. *Gut*.

[10] Nielsen O. H., Vainer B., Rask-Madsen J. (2008). Non-IBD and noninfectious colitis. *Nature Clinical Practice Gastroenterology and Hepatology*.

[11] Neut C., Colombel J. F., Guillemot F. (1989). Impaired bacterial flora in human excluded colon. *Gut*.

[12] Villanacci V., Talbot I. C., Rossi E., Bassotti G. (2007). Ischaemia: A pathogenetic clue in diversion colitis?. *Colorectal Disease*.

[13] Guillemot F., Colombel J. F., Neut C. (1991). Treatment of diversion colitis by short-chain fatty acids - Prospective and double-blind study. *Diseases of the Colon & Rectum*.

[14] Harig J. M., Soergel K. H., Komorowski R. A., Wood C. M. (1989). Treatment of diversion colitis with short-chain-fatty acid irrigation. *The New England Journal of Medicine*.

[15] Pacheco R. G., Esposito C. C., Müller L. C. M. (2012). Use of butyrate or glutamine in enema solution reduces inflammation and fibrosis in experimental diversion colitis. *World Journal of Gastroenterology*.

[16] Alvarenga V., Pacheco R. G., Esposito C. C. (2014). Ascidian (chordate-tunicate) and mammalian heparin enemas attenuate experimental diversion colitis. *Surgery (United States)*.

[17] Keli E., Bouchoucha M., Devroede G., Carnot F., Ohrant T., Cugnenc P.-H. (1997). Diversion-related experimental colitis in rats. *Diseases of the Colon and Rectum*.

[18] Martinez C. A. R., de Campos F. G. C. M., de Carvalho V. R. J. (2015). Claudin-3 and occludin tissue content in the glands of colonic mucosa with and without a fecal stream. *Journal of Molecular Histology*.

[19] Babickova J., Tothova L., Lengyelova E., Bartonova A., Hodosy J., Gardlik R. (2015). Sex Differences in Experimentally Induced Colitis in Mice: a Role for Estrogens. *Inflammation*.

[20] Zantl N., Uebe A., Neumann B. (1998). Essential role of gamma interferon in survival of colon ascendens stent peritonitis, a novel murine model of abdominal sepsis. *Infection and Immunity*.

[21] Beyer K., Stollhof L., Poetschke C. (2016). TNF-related apoptosis-inducing ligand deficiency enhances survival in murine colon ascendens stent peritonitis. *Journal of Inflammation Research*.

[22] Mello R. D. O., da Silva C. M. G., Fonte F. P. (2012). Evaluation of the number of goblet cells in crypts of the colonic mucosa with and without fecal transit. *Revista do Colegio Brasileiro de Cirurgioes*.

[23] Kabir S. I., Kabir S. A., Richards R., Ahmed J., MacFie J. (2014). Pathophysiology, clinical presentation and management of diversion colitis: A review of current literature. *International Journal of Surgery*.

[24] Lu E. S., Lin T., Harms B. L., Gaumnitz E. A., Singaram C. (1995). A severe case of diversion colitis with large ulcerations. *American Journal of Gastroenterology*.

[25] Boyce S. A., Hendry W. S. (2008). Diversion colitis presenting with massive rectal distension and bilateral ureteric obstruction. *International Journal of Colorectal Disease*.

[26] Szczepkowski M., Banasiewicz T., Kobus A. (2017). Diversion colitis 25 years later: the phenomenon of the disease. *International Journal of Colorectal Disease*.

[27] WARREN B. F., SHEPHERD N. A. (1992). Diversion proctocolitis. *Histopathology*.

[28] Valcz G., Krenács T., Sipos F. (2011). Lymphoid aggregates may contribute to the migration and epithelial commitment of Bone marrow-derived cells in colonic mucosa. *Journal of Clinical Pathology*.

[29] Sipos F., Muzes G., Galamb O. (2010). The possible role of isolated lymphoid follicles in colonic mucosal repair. *Pathology and Oncology Research*.

[30] Komorowski R. A. (1990). Histologic spectrum of diversion colitis. *The American Journal of Surgical Pathology*.

[31] Gálvez J., Garrido M., Merlos M., Torres M. I., Zarzuelo A. (2000). Intestinal anti-inflammatory activity of UR-12746, a novel 5-ASA conjugate, on acute and chronic experimental colitis in the rat. *British Journal of Pharmacology*.

[32] Kissmeyer-Nielsen P., Christensen H., Laurberg S. (1994). Diverting colostomy induces mucosal and muscular atrophy in rat distal colon. *Gut*.

[33] Yan S. L. S., Russell J., Harris N. R., Senchenkova E. Y., Yildirim A., Granger D. N. (2013). Platelet abnormalities during colonic inflammation. *Inflammatory Bowel Diseases*.

[34] Fajardo G., HÖrnicke H. (1989). Problems in estimating the extent of coprophagy in the rat. *British Journal of Nutrition*.

[35] Ebino K. Y., Yoshinaga K., Suwa T., Kuwabara Y., Takahashi K. W. (1989). Effects of prevention of coprophagy on pregnant mice—is coprophagy beneficial on a balanced diet?. *Jikken dobutsu. Experimental animals*.

[36] Tremaroli V., Bäckhed F. (2012). Functional interactions between the gut microbiota and host metabolism. *Nature*.

[37] Krajmalnik-Brown R., Ilhan Z.-E., Kang D.-W., DiBaise J. K. (2012). Effects of gut microbes on nutrient absorption and energy regulation. *Nutrition in Clinical Practice*.

